# Independent external validation of the foramen magnum meningioma risk score: limited discrimination and overestimation of absolute risk

**DOI:** 10.3389/fneur.2026.1926742

**Published:** 2026-10-23

**Authors:** Nafiye Sanlier, Umid Sulaimanov, Ufuk Erginoglu, Behman Demir, Ahmed Rasim Bayramoglu, Mariagrazia Nizzola, Abdullah Keles, Mustafa K. Baskaya

**Affiliations:** Department of Neurological Surgery, School of Medicine and Public Health, University of Wisconsin-Madison, Madison, WI, United States

**Keywords:** discrimination, external validation, foramen magnum meningioma, model calibration, postoperative complications, prediction model, skull base surgery, surgical risk score

## Abstract

**Objective:**

Foramen magnum meningiomas (FMMs) cause substantial operative morbidity because of their close relationship to the brainstem, lower cranial nerves, vertebral artery (VA), and upper cervical spinal cord. The Foramen Magnum Meningioma Risk Score (FMMRS) is the only published preoperative, MRI-based model for estimating postoperative complication risk. However, it has not been independently validated, and its calibration is unknown. We performed independent external validation, focusing on absolute risk transportability.

**Methods:**

In this single-center, retrospective study, 40 consecutive adults who underwent microsurgical FMM resection were analyzed. The FMMRS was recalculated *de novo* from preoperative MRI by a neurosurgeon blinded to outcomes, with independent re-scoring by a second neurosurgeon. The primary outcome was any postoperative complications; a major complication was a prespecified secondary outcome. Discrimination was assessed using the area under the receiver operating characteristic curve (AUC) and calibrated the primary outcome using the calibration slope and intercept, the Brier score, and observed-vs-expected rates across the published risk groups.

**Results:**

Complications occurred in 14/40 patients (35.0%) with major complications in 7/40 (17.5%). Point estimates suggested limited discrimination for both endpoints (AUCs of 0.64 [95% CI 0.46–0.79] and 0.67 [95% CI 0.48–0.84]), with confidence intervals that did not exclude chance. Observed complication rates were lower than predicted in the intermediate- and high-risk groups, indicating an overestimation of absolute risk. The calibration slope was 0.23 (95% CI − 0.04 to 0.57), the intercept −0.90 (95% CI − 1.45 to −0.57), and the Brier score (0.333) exceeded that of the null model (0.228). The largest discrepancy was in the high-risk group, where the expected rate was 96.4% vs. an observed rate of 42.9% (95% CI 21.4–67.4%). In exploratory threshold analysis, FMMRS ≥6 captured all major complications, whereas the published high-risk threshold of ≥8 missed approximately half.

**Conclusion:**

In this independent external cohort, the FMMRS retained only limited value as an ordinal stratifier for complication risk and overestimated absolute complication probabilities, particularly for high-risk patients. Its published probabilities should not guide individualized preoperative counseling without recalibration; larger multicenter studies with patient-level data are needed to refine and validate a clinically reliable preoperative prediction model.

## 1 Introduction

Foramen magnum meningiomas (FMMs) account for 0.3–3.2% of all intracranial meningiomas; however, they carry a surgical risk disproportionate to their rarity, given their intimate relationship with the brainstem, lower cranial nerves, the vertebral artery and its branches, and the upper cervical cord (1–3). Due to their slow growth, they typically reach considerable size before becoming symptomatic, presenting with long-tract signs, lower cranial nerve deficits, and occipitocervical pain (4–6).

FMMs are among the most surgically challenging skull-base meningiomas (7–10). Maximal safe resection remains the primary surgical objective, but when adherence to critical neurovascular structures precludes safe complete removal, residual tumors can be managed with surveillance or adjuvant radiosurgery (3, 10). Because anticipated morbidity informs preoperative counseling and planning, a reproducible, image-based tool to estimate surgical risk is clinically valuable (11, 12).

FMM resection carries substantial morbidity, with contemporary series reporting complication rates ranging from approximately 25% to more than 60% (7, 11, 13). Common complications include lower cranial nerve dysfunction, dysphagia, cerebrospinal fluid (CSF) leak, pseudomeningocele, hydrocephalus, and vertebral artery (VA) injury (13–16). These complications are not randomly distributed. A few tumor features assessable on preoperative imaging are repeatedly associated with higher complication rates across independent surgical series, including anterior location relative to the dentate ligament, craniospinal extension, and encasement of the vertebral artery or its branches. Because these features are apparent before surgery and consistent across studies, they provide a rational basis for a preoperative, imaging-based risk score (5, 9, 17).

Several classification systems have been proposed to characterize the anatomical heterogeneity of FMMs and guide surgical planning. The Bruneau–George classification (based on tumor compartment and dural attachment), the Li et al. system (incorporating tumor–brainstem relationships), the more recent Gattozzi et al. system (based on topographic position relative to the neurovascular bundle), and other reports have provided useful anatomical and operative frameworks, particularly for approach selection (8, 9, 18–20). However, these systems were not developed to estimate individualized postoperative complication risk (8, 18–20). The unmet need is therefore not another descriptive classification but independent validation of an image-based risk score that estimates complication risk before surgery.

To date, the Foramen Magnum Meningioma Risk Score (FMMRS) is the only published score designed to quantify postoperative complication risk from preoperative MRI alone (3). Derived from a multi-institutional cohort of 51 patients, it integrates craniocaudal location, anteroposterior location relative to the dentate ligament, and VA involvement into an integer score of 2 to 9, stratifying patients into low-, intermediate-, and high-risk groups. It achieved an area under the receiver operating characteristic curve (AUC) of 0.81 in the derivation cohort and 0.86 in the same study’s small external test set (*n* = 15, separate from the 51-patient derivation cohort) from a contributing institution (3). Two limitations temper its immediate clinical use. First, although the authors reported both internal and external validation, they acknowledged that this external validation set was small (*n* = 15) and that larger studies were required (3). That validation was also not fully independent, as it was performed within the original investigator group, so a truly independent assessment by an unrelated team remained lacking. Second, validation addressed discrimination without formal calibration. A key distinction is that a model may rank patients correctly by risk while still misestimating absolute probabilities in a new population (3). For a score intended to guide counseling, the transportability of predicted risks is essential (21).

We performed independent, single-center external validation of the FMMRS in a consecutive surgical cohort. The primary objective was to assess discrimination for both any postoperative complication and major complication and thus to evaluate calibration of the published absolute-risk estimates for any postoperative complication. Secondary analyses examined observed complication rates across the published risk groups and the exploratory performance of alternative FMMRS thresholds.

## 2 Methods

### 2.1 Study design and patient cohort

This study is a single-center, retrospective external validation of the FMMRS, originally developed by Fatima et al. (3). Patients who underwent surgery between 2006 and 2025 were screened. Forty-two patients with foramen magnum meningiomas were assessed for eligibility, excluding 2 because their preoperative MRIs were inadequate for FMMRS component coding, yielding a final cohort of 40 patients. The study was conducted in accordance with the Declaration of Helsinki, applicable local regulations, and the TRIPOD reporting guidelines. The study was approved by the Institutional Review Board of the University of Wisconsin-Madison (protocol 2024-1867-CP001), and all patient identifiers were removed before analysis.

Consecutive adult patients (≥18 years) who underwent microsurgical resection for FMM by a single surgeon at our institution were identified retrospectively. Inclusion criteria were: histopathologically confirmed meningioma located within the foramen magnum region as defined by the anatomical boundaries used in the original FMMRS study, availability of preoperative MRI permitting coding of FMMRS components, and availability of operative data. Excluded were patients treated with radiotherapy or stereotactic radiosurgery alone, radiation-induced lesions, tumors with an epicenter outside the foramen magnum, or multiple meningiomas (other than a solitary foramen magnum meningioma in the setting of neurofibromatosis type 2). No formal sample-size calculation was performed because all consecutively treated eligible patients during the study period were included. Given the limited number of outcome events, the validation estimates were interpreted as preliminary and hypothesis-generating.

### 2.2 Recalculation of FMMRS and expected risk

The FMMRS is a 2–9 point score composed of three preoperative imaging variables, weighted according to the FMMRS scheme (Supplementary Table S1) adapted from Fatima et al. (3) (Table 1). In the present cohort, FMMRS was recalculated *de novo* using preoperative MRI analysis for each patient, independent of any prior assessment in the clinical record. The most recent preoperative contrast-enhanced MRI was reviewed for FMMRS coding, with the FMMRS calculated from three MRI-based variables: anteroposterior location relative to the dentate ligament, craniocaudal extension, and VA involvement. Posterior-to-dentate tumors received 1 point and anterior tumors 5 points. Spino-cranial tumors received 1 point, and craniospinal tumors 3 points (based on craniocaudal extension, craniospinal tumors are mainly intracranial, with caudal extension through the foramen magnum into the spinal canal, whereas spino-cranial tumors are mainly intraspinal, with rostral extension into the cranial cavity) (3). VA involvement was scored as 0 when absent and 1 when contact, abutment, displacement, compression, or encasement was present. Total scores ranged from 2 to 9 and were grouped into low (2–4), intermediate (5–7), and high risk (8, 9).

**Table 1:** Observed and expected complication rates by the FMMRS risk group for each outcome.

Risk group	*n*	Expected %	Expected band %	Observed n/N	Observed % (95% CI)	Δ (pp)
*Primary endpoint (any postoperative complication)*						
Low (2–4)	6	8.94	4.4–13.5	1/6	16.67(3.0–56.4)	+7.72
Intermediate (5–7)	20	59.37	52.3–66.4	7/20	35.00 (18.1–56.7)	−24.37
High (8–9)	14	96.37	93.4–99.3	6/14	42.86 (21.4–67.4)	−53.51
*Major complication*						
Low (2–4)	6	–	–	0/6	0.00 (0.0–39.0)	–
Intermediate (5–7)	20	–	–	3/20	15.00 (5.2–36.0)	–
High (8–9)	14	–	–	4/14	28.57 (11.7–54.6)	–

Expected % is the mean of the FMMRS score-specific midpoint probabilities (derivation study, Fatima et al., their table) for the patients in that risk group, reflecting the actual score composition within each stratum. The expected band is the mean of the corresponding published lower and upper risk bounds. Δ values express the absolute difference between observed and expected rates in percentage points. Expected rates and Δ are reported for the primary endpoint only; for major complication, only observed rates with 95% Wilson confidence intervals are shown. FMMRS, Foramen Magnum Meningioma Risk Score; CI, confidence interval; pp, percentage points.

Expected complication probabilities were obtained at the score level from the published FMMRS risk bands (Supplementary Table S1; from Fatima et al. (3) Table 1) and assigned to each patient based on their recalculated FMMRS total. The midpoints of the score-specific probability ranges were used, with values ranging from 2.38 to 98.05% for scores 2 through 9. Risk-group-level expected probabilities were calculated as the mean of the patient-level estimates within the corresponding group, preserving the actual score composition of each stratum. The full lower and upper bounds of each score’s published band were also retained at the patient level for the band-based calibration robustness analysis described below. Patient-level FMMRS components, published risk estimates, and observed outcomes are provided in Supplementary Table S2. MRI image classifications were performed independently by two neurosurgeons blinded to postoperative outcomes. The inter-rater agreement was substantial and near-perfect (Cohen’s κ 0.82–1.00), with 6 borderline cases involving VA involvement and the CS/SC distinction, resolved by consensus.

### 2.3 Outcomes and variables

Two complication outcomes were prespecified. The primary outcome, any postoperative complication (AnyComp), is a composite outcome defined as any postoperative adverse event attributable to surgery and consistent with the complication categories reported in the FMMRS derivation cohort, including a new or worsened neurological deficit, lower cranial nerve dysfunction or dysphagia (transient or persistent), CSF-related complication (CSF leak, hydrocephalus requiring shunting), and aspiration pneumonia. Patients with more than one complication were counted once for the binary AnyComp endpoint, with individual complications recorded separately for descriptive analyses. The secondary outcome, or major complication, was a secondary, investigator-defined endpoint specified for this study rather than part of the original FMMRS derivation. Accordingly, it was analyzed descriptively and was not subjected to calibration. It was defined as an AnyComp event that resulted in a persistent neurological deficit at ≥3-month follow-up, that required reoperation or permanent enteral feeding access for postoperative dysphagia, or led to complication-related perioperative mortality (3, 9, 11, 19). Complications were considered postoperative if they occurred during the perioperative period and were directly attributable to the index operation. Delayed events related to tumor progression, adjuvant treatment, or unrelated medical conditions were not classified as postoperative complications.

Patient-level demographic, clinical, radiological, surgical, and outcome variables were extracted from the electronic medical record and from a prospectively maintained surgical database. Recorded variables included age, sex, presenting symptoms (neck pain, headache, hemiparesis, ataxia, imbalance, nausea/vomiting, paresthesia, and cranial nerve deficits), preoperative Karnofsky Performance Status (KPS), tumor volume (cm^3^), WHO grade, craniospinal orientation, relationship to the dentate ligament, VA involvement, brainstem and/or cervical cord compression, extent of resection, length of stay, postoperative symptomatic course, and follow-up duration. Postoperative symptomatic improvement was defined as documented improvement of preoperative neurological symptoms on the treating clinician’s assessment at the last follow-up, recorded as improved, stable, or worsened. Variables from Table 2 of the derivation study (3) were used as a reference for case-mix comparison. These variables were collected to characterize the validation cohort and compare its case mix with the derivation cohort; they were not entered into model updating or multivariable analyses.

**Table 2:** Comparative characteristics of the present external validation cohort and the FMMRS derivation cohort.

Characteristic	Present cohort (*n* = 40)	FMMRS cohort (*n* = 51)	*p*	SMD
Patient characteristics				
Age, years–median (range)	56.5 (29–78)	59 (20–77)	–^a^	–^a^
Female sex–*n* (%)	27 (67.5)	38 (74.5)	0.49	−0.15
Presenting KPS–median (range)	80 (40–90)	70 (50–80)	–^a^	–^a^
Follow-up, months–median (range)	39.5 (3–192)	36 (0.3–180)	–^a^	–^a^
Presenting symptoms–*n* (%)				
Neck pain	22 (55.0)	41 (80.4)	**0.012**	−0.56
Headache	20 (50.0)	39 (76.5)	**0.014**	−0.57
Hemiparesis	11 (27.5)	30 (58.8)	**0.003**	−0.67
Ataxia	4 (10.0)	25 (49.0)	**<0.001**	−0.95
Imbalance	10 (25.0)	24 (47.1)	**0.049**	−0.47
Nausea/vomiting	3 (7.5)	22 (43.1)	**<0.001**	−0.90
Cranial nerve deficit	19 (47.5)	14 (27.5)	0.078	0.42
Tumor characteristics				
Tumor volume, cm^3^–median (range)	5.0 (0.3–33.1)	8.18 (1.77–57.9)	–^a^	–^a^
WHO Grade I–*n* (%)	34 (85.0)	47 (92.2)	0.33^b^	−0.23^b^
WHO Grade II/III–*n* (%)	6 (15.0)	4 (7.8)	0.33^b^	0.23^b^
Craniospinal orientation–*n* (%)	20 (50.0)	34 (66.7)	0.13	−0.34
Anterior to dentate ligament–*n* (%)	30 (75.0)	37 (72.5)	0.82	0.06
Vertebral artery involvement–*n* (%)	30 (75.0)	27 (52.9)	**0.049**	0.47
Brainstem/spinal cord compression–*n* (%)	36 (90.0)	47 (92.2)	0.73	−0.08
Surgical outcomes –present cohort only^c^				
Gross total resection (no residual on 72-h MRI)–*n* (%)	31 (77.5)	–	–	–
Length of stay, days–median (IQR)	4 (3–8)	–	–	–
Symptomatic improvement–*n* (%)	34 (85.0)	–	–	–
Perioperative mortality–*n* (%)	0 (0)	–	–	–
FMMRS distribution–present cohort only^c^				
FMMRS score–median (IQR)	7 (6–9)	–	–	–
Low risk (2–4)–*n* (%)	6 (15.0)	–	–	–
Intermediate risk (5–7)–*n* (%)	20 (50.0)	–	–	–
High risk (8–9)–*n* (%)	14 (35.0)	–	–	–

Continuous variables are presented as median (range or IQR as indicated) and categorical variables as *n* (%). *p*-values are derived from Fisher’s exact test for binary categorical comparisons. p-values ≤0.05 indicate statistical significance. Boldface marks statistically significant values. Symptom comparisons are unadjusted for multiple testing. SMD is reported for binary comparisons only, with values >0.3 indicating a non-negligible between-cohort imbalance. IQR, Interquartile Range; KPS, Karnofsky Performance Status; WHO, World Health Organization; FMMRS, Foramen Magnum Meningioma Risk Score; SMD, standardized mean difference.

a Comparison not performed because patient-level data were not available for the derivation cohort; these variables are compared descriptively.

b WHO Grade I and Grade II/III are complementary categories of a single binary comparison, so the p-value is shared, and the standardized mean differences are equal in magnitude and opposite in sign.

c Not applicable; these variables were not reported in the derivation cohort.

### 2.4 Statistical analysis

Statistical analyses were performed in Python (version 3.12; Python Software Foundation). No data were missing for the FMMRS components, study outcomes, or covariates. Continuous variables were summarized as median (IQR or range) and categorical variables as n (%). Categorical variables were compared using Fisher’s exact test; continuous variables with available patient-level data were compared using the Mann–Whitney U test. 95% confidence intervals for proportions were calculated using the Wilson method. Comparisons of continuous variables with that cohort are presented descriptively, because patient-level individual data were not available for the derivation cohort. For case-mix comparisons between the present cohort and the FMMRS derivation cohort, binary variables with published counts were analyzed using Fisher’s exact test. These variables included sex, WHO ≥ II, anterior location relative to the dentate ligament, craniospinal orientation, VA involvement, brainstem/medullary compression, and presenting symptoms. Hypothesis tests based on the published aggregate data were interpreted cautiously and were not used to infer cohort equivalence. Standardized mean differences (SMD) were also calculated as a sample-size-independent measure of imbalance.

Discrimination of the FMMRS was assessed using the area under the receiver operating characteristic curve (AUC; c-statistic). The FMMRS was treated as an ordinal predictor, and the outcomes as binary variables; the primary complication outcomes were analyzed separately. 95% confidence intervals for the AUC were obtained by stratified bootstrap resampling with 10,000 iterations. Pointwise 95% confidence bands for the ROC curves were derived from the same stratified bootstrap procedure. Calibration analyses were restricted to the primary AnyComp endpoint, because the published FMMRS risk estimates were derived for overall postoperative complications rather than for major complications. For the secondary major-complication endpoint, we evaluated discrimination, observed event rates by risk group, threshold operating characteristics, and monotonic trend only. Because the derivation study reported score-specific probability intervals rather than an individual-level regression equation, these analyses assessed the transportability of the published score-to-risk mapping rather than the recalibration of the original model equation. Point-probability calibration metrics were therefore calculated using published score-specific midpoint probabilities. These midpoint-based analyses included calibration slope and intercept, Brier score, and the Hosmer-Lemeshow goodness-of-fit test at the risk-group level. Confidence intervals for the calibration slope and intercept were obtained using the same 10,000-iteration stratified bootstrap procedure used for the AUC. The calibration model was fit as logit(P[Y = 1]) = α + β·logit(p̂), where p̂ is the patient-level, score-specific predicted probability derived from published FMMRS risk bands (Supplementary Table S1; from Fatima et al. (3) Table 1). The Hosmer–Lemeshow test was computed across the three published risk groups; expected event counts were based on the mean of patient-level predicted probabilities within each group. Because this statistic is inflated and unstable with a few risk groups and near-certain predicted probabilities, the calibration slope and observed-vs-expected comparison were treated as primary calibration evidence. The derivation study reported predicted risk as score-specific probability bands rather than point estimates. A band-based robustness check was therefore performed: for each risk group, the published lower and upper bounds were averaged across its patients, and the observed rate (95% Wilson interval) was compared against the resulting band. Observed and expected event rates by risk group, and the absolute difference between them (Δ), are reported in percentage points. A leave-one-out case-deletion analysis was performed to assess whether any single observation materially influenced AnyComp discrimination and calibration estimates.

Monotonic trends in observed event rates across FMMRS score levels (2–9) were assessed using the Cochran-Armitage trend test. The Z statistic and two-sided *p*-value are reported, with significance set at α = 0.05. For each possible FMMRS threshold (≥3 through ≥9), sensitivity, specificity, positive predictive value (PPV), negative predictive value (NPV), accuracy, and the Youden index were calculated separately for each outcome. The threshold that maximized the Youden index was designated the optimal cut-point; the published risk-group thresholds of ≥5 and ≥8 are also reported as *a priori* clinical thresholds. A neighboring lower threshold was designated sensitivity-oriented when its Youden index was within 1 percentage point of the maximum, and its sensitivity was higher. All threshold and cut-point analyses were regarded as exploratory and hypothesis-generating. To reduce single-pipeline computation errors, core metrics were verified in a second, independent computation pass using complementary methods. AUC point estimates differed by <0.001 across passes. Because the accrual period spanned two decades, a *post hoc* analysis compared case mix and outcomes between the first and second halves of the period, defined as 2006 to 2015 and 2016 to 2025. This analysis was exploratory and was not prespecified.

## 3 Results

### 3.1 Cohort characteristics and postoperative complications

Forty consecutive surgically treated FMM patients from this single-surgeon series were included (Table 2). Median age was 56.5 (range 29–78), with 67.5% women, and the median presenting KPS was 80. WHO Grade I tumors accounted for 85.0% of cases; median tumor volume was 5.0 cm^3^. Anatomically, 50.0% of tumors extended craniospinally, 75.0% were located anterior to the dentate ligament, and 75.0% involved the VA. Gross total resection was achieved in 77.5% of patients, with a 4-day median length of stay. Postoperative symptomatic improvement was observed in 85.0% of patients. Median follow-up was 39.5 months. The median FMMRS was 7, with patients distributed across the low-, intermediate-, and high-risk groups (*n* = 6, *n* = 20, and *n* = 14, respectively).

Compared with the FMMRS derivation cohort, the present cohort had more frequent VA involvement (75.0% vs. 52.9%; SMD 0.47) and lower frequencies of several presenting symptoms other than cranial nerve deficits (neck pain, headache, hemiparesis, ataxia, imbalance, and nausea/vomiting). Median tumor volume was lower in the present cohort (5.0 vs. 8.18 cm^3^), and presenting KPS was higher (80 vs. 70), although formal comparison of these continuous variables was not possible because patient-level derivation data were unavailable (Table 2).

Two complication outcomes were analyzed in this validation. The primary outcome, any postoperative complication (AnyComp), was observed in 14/40 patients (35.0%). The major complication outcome occurred in 7/40 patients (17.5%). The composition of complication events by type and the criteria met for major complications are detailed in Table 3. There was no perioperative mortality. Of the 14 patients who experienced any postoperative complication, 10 (71.4%; 95% CI 45.4–88.3) showed improvement in their preoperative neurological symptoms at the last follow-up.

**Table 3:** Composition of postoperative complication events by type and major-complication criterion (*n* = 40).

Complication/Criterion	AnyComp events (*n* = 14)	Major events (*n* = 7)
Complication type		
CSF leak	2	2
Swallowing dysfunction (CN IX–X)	5	5
Hydrocephalus requiring shunt	2	2
Imbalance and ataxia	1	0
Meningitis	0	0
Aspiration pneumonia	4	3
Quadriparesis	0	0
CN VI palsy	2	0
CN VII palsy	1	0
CN VIII palsy	0	0
CN XI palsy	1	1
Transient lower cranial nerve deficit	10	4
Persistent lower cranial nerve deficit	2	2
Major-complication criterion		
Reoperation	–	5
Permanent feeding-tube placement (PEG)	–	5
Persistent neurological deficit at ≥3 months	–	2
Perioperative mortality	–	0

Counts reflect the number of patients in whom each item was recorded. A single patient may contribute more than one complication or meet more than one major criterion, so column totals exceed the 14 any-complication and 7 major-complication patients. For the major-complication criteria, the any-complication column is not applicable (−). In the type rows, the Major column reflects co-occurrence, not a major-defining criterion. CSF, cerebrospinal fluid; CN, cranial nerve; PEG, percutaneous endoscopic gastrostomy.

### 3.2 Discrimination and calibration

Point estimates suggested limited FMMRS discrimination for the primary and major outcomes, with wide confidence intervals that did not exclude chance (Figure 1). The AUC for any complication was 0.64 (95% CI 0.46–0.79). For major complications, the AUC was 0.67 (95% CI 0.48–0.84).

**Figure 1:**
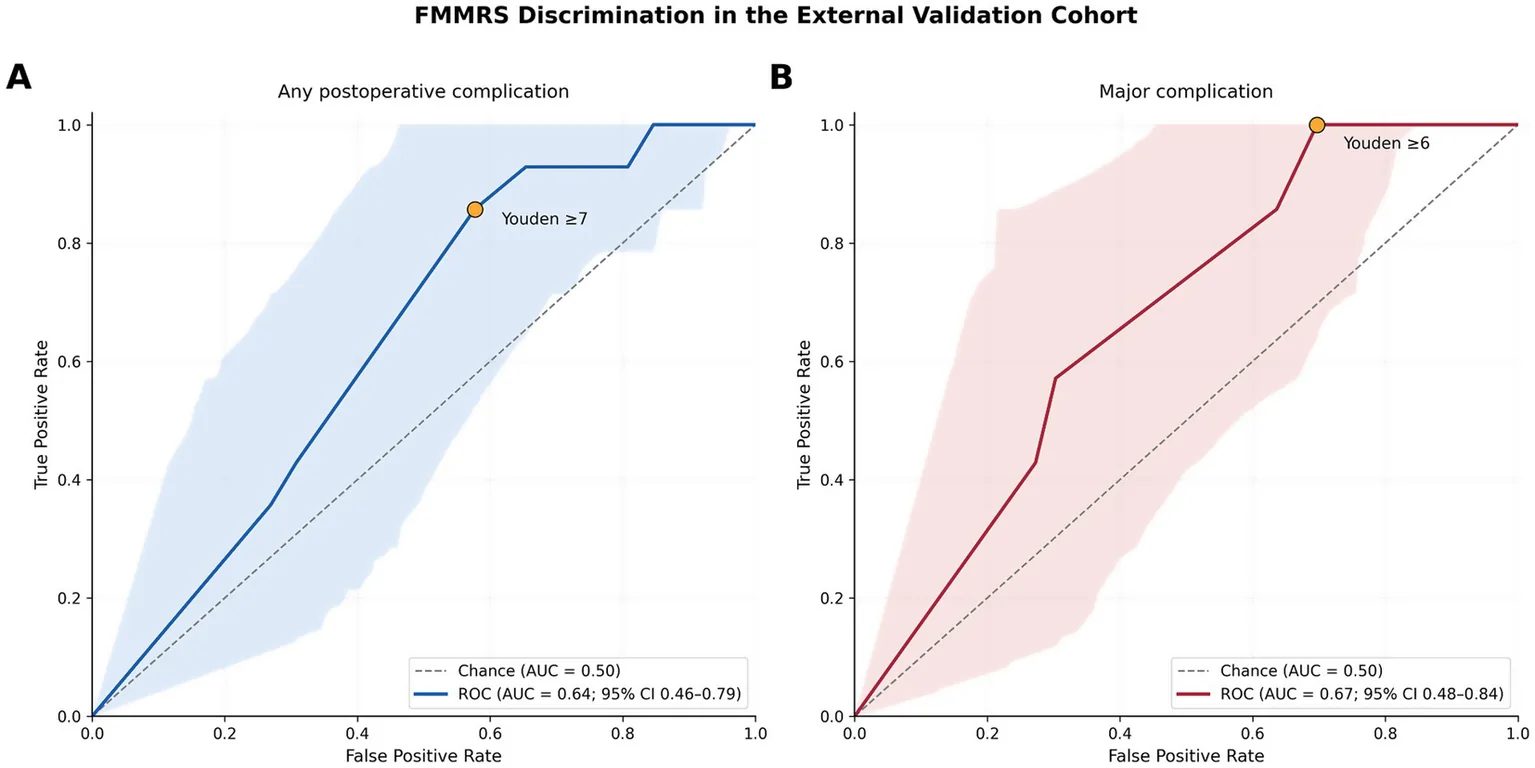
ROC curves illustrate FMMRS discrimination in the external validation cohort. **(A)** Primary outcome (any postoperative complication); **(B)** secondary outcome (major complication). Shaded areas represent 95% confidence intervals obtained by stratified bootstrap with 10,000 iterations; the Youden-optimal cut-point is marked on each panel (Panel **A**: FMMRS ≥7; Panel **B**: FMMRS ≥6). The dashed line represents chance (AUC = 0.50). AUC, area under the receiver operating characteristic curve; CI, confidence interval; FMMRS, Foramen Magnum Meningioma Risk Score; ROC, receiver operating characteristic.

Calibration of the primary endpoint was suboptimal; predicted probabilities exceeded observed event rates in the intermediate- and high-risk groups (Table 4). The calibration slope and intercept were 0.23 (95% CI −0.04 to 0.57) and −0.90 (95% CI −1.45 to −0.57) for any complication. The largest expected-observed discrepancy occurred in the high-risk group, where the expected probability was 96.4% vs. an observed event rate of 42.9% (Δ = −53.5). At the individual FMMRS score level, observed event rates were generally lower than the published estimates at scores 5 through 9, although these score-level estimates were imprecise because of sparse cells (Figure 2B). The Brier score for any complication was 0.333, exceeding the null-model value of 0.228 (the overall event rate applied to every patient) and indicating no improvement over the base rate. The Hosmer–Lemeshow test was reported for completeness only; given the three risk groups and near-certain high-risk predicted probabilities, this statistic is inflated and unstable (χ^2^ = 118.54; df = 1; *p* < 0.0001). In leave-one-out analyses, discrimination and calibration were stable across all 40 iterations (AnyComp AUC 0.62–0.67; calibration slope 0.20–0.29); estimates were not materially altered by deletion of any individual case (Figure 3).

**Table 4:** Discrimination, calibration, and trend metrics of the FMMRS for both outcomes.

Metric	Any complication	Major complication
Number of events, *n* (%)	14 (35.0)	7 (17.5)
AUC (95% CI)	0.64 (0.46–0.79)	0.67 (0.48–0.84)
Calibration slope (95% CI)	0.23 (−0.04 to 0.57)	–
Calibration intercept (95% CI)	−0.90 (−1.45 to −0.57)	–
Brier score	0.333 (null-model 0.228)	–
Cochran-Armitage trend Z (p)	1.66 (0.097)	1.54 (0.124)

Confidence intervals for the AUC, calibration slope, and calibration intercept were obtained using 10,000 bootstrap resamples. Calibration slope and intercept are derived from a patient-level logistic calibration model. Null-model Brier (0.228) assigns the overall event rate to every patient. AUC, area under the receiver operating characteristic curve; CI, confidence interval.

**Figure 2:**
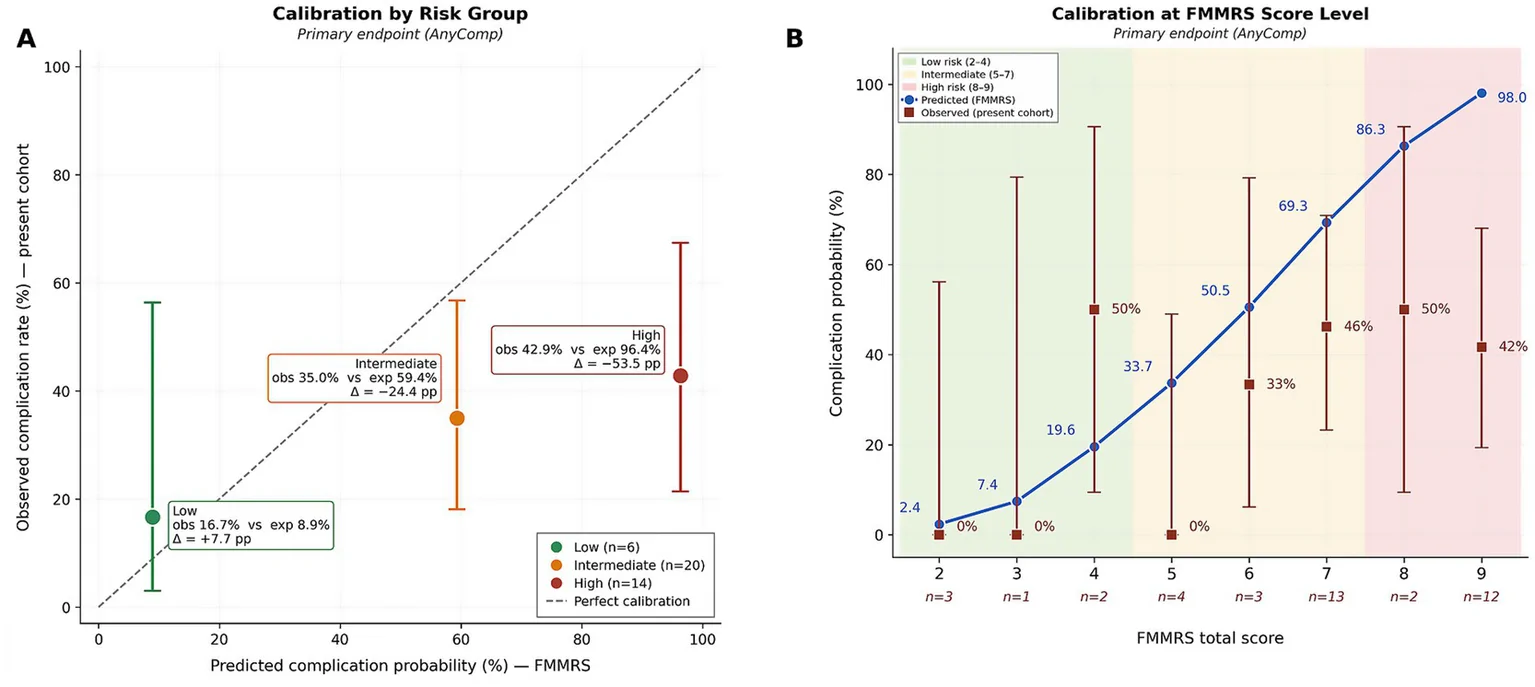
FMMRS calibration for the primary endpoint (any postoperative complications). **(A)** Risk-group level: observed event rates in the present cohort plotted against patient-level mean predicted probabilities derived from the published FMMRS score-specific midpoint estimates; error bars represent 95% Wilson confidence intervals, and Δ indicates observed−expected difference in percentage points. The dashed line represents perfect calibration (y = x). **(B)** Score-level calibration: published FMMRS predicted probabilities are shown as a blue line and observed event rates in the present cohort are shown as red squares with 95% Wilson confidence intervals at each integer score; per-score n is shown below the x-axis, and background shading denotes the published risk-group categories: low 2–4, intermediate 5–7, and high 8–9. AnyComp, any postoperative complication; CI, confidence interval; exp., expected; FMMRS, Foramen Magnum Meningioma Risk Score; obs, observed; pp., percentage points; Δ, observed−expected difference.

**Figure 3:**
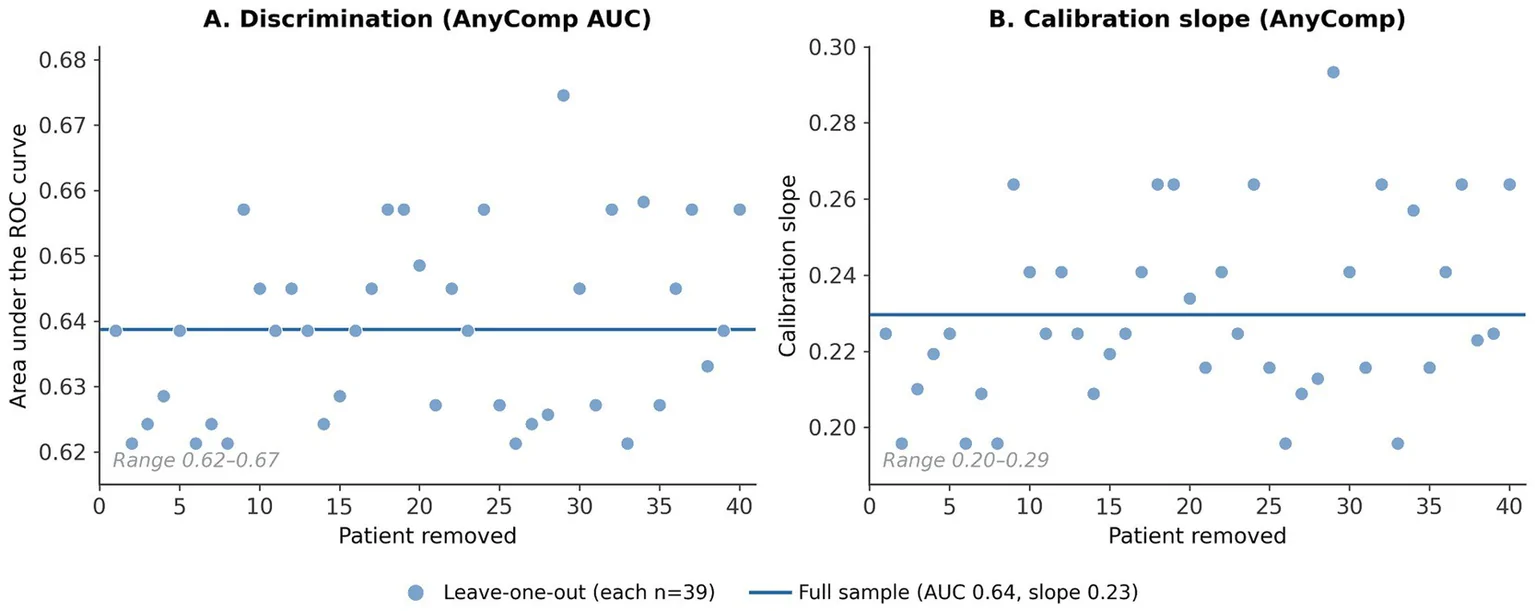
Leave-one-out stability of FMMRS discrimination and calibration. Each point shows the metric re-estimated after removing one patient (40 iterations, each *n* = 39). **(A)** Any-complication AUC. **(B)** Any-complication calibration slope. The horizontal line is the full-sample estimate. No single patient drove the results; both metrics remained within a narrow range across all 40 iterations (AUC 0.62–0.67, calibration slope 0.20–0.29). FMMRS, Foramen Magnum Meningioma Risk Score; AUC, area under the ROC curve.

Because the cohort accrued over two decades, outcomes were compared between the first and second halves of the accrual period (Supplementary Table S4). The distribution of FMMRS scores was comparable across the two periods, but any postoperative complication occurred in 11 of 16 patients (68.8%) treated between 2006 and 2015 and in 3 of 24 (12.5%) treated between 2016 and 2025. Within the high-risk group, observed rates fell 29.4 percentage points below the published expectation in the earlier period and 71.6 percentage points below it in the later period. Era-stratified discrimination and calibration were not estimated because only three primary events occurred in the later period.

### 3.3 Risk group and threshold performance

Observed AnyComp rates increased numerically across the published FMMRS risk groups. Compared with published expectations, observed rates were lower in the intermediate- and high-risk groups, with the largest discrepancy in the high-risk group (Table 1, Figure 2A). Expected event rates were calculated as the mean of the published score-specific midpoint probabilities (3) for the patients within each risk group. For any complication, the low-risk group performed close to the patient-level expectation (observed 16.7%; expected 8.9%; Δ + 7.7), whereas both the intermediate-risk group (35.0% vs. 59.4%; Δ − 24.4) and especially the high-risk group (42.9% vs. 96.4%; Δ − 53.5) were overpredicted. In both the intermediate- and high-risk groups, the observed rate fell below the lower bound of the published expected band (Table 1), and in the high-risk group, its entire 95% confidence interval lay below that bound. For major complications, the observed rates were 0.0% (95% CI 0.0–39.0), 15.0% (95% CI 5.2–36.0), and 28.6% (95% CI 11.7–54.6) in the low-, intermediate-, and high-risk groups, respectively (Table 1). The Cochran-Armitage trend test was not statistically significant for either outcome (any complication: Z = 1.66; *p* = 0.097; major complication: Z = 1.54; *p* = 0.124), although it was underpowered to detect a monotonic trend at this sample size.

Because event counts were limited, the following threshold analyses are exploratory. For any postoperative complication, the Youden-optimal threshold was FMMRS ≥7 (Youden +28.0; sensitivity 85.7%; specificity 42.3%; NPV 84.6%). FMMRS ≥6 yielded a comparable Youden value (+27.5) while offering higher sensitivity (92.9%) and NPV (90.0%). For major complications, the Youden-optimal threshold was FMMRS ≥6 (Youden +30.3), identifying all 7 major complications, with sensitivity 100% (95% CI 65–100), specificity 30.3%, and NPV 100% (95% CI 72–100). The published high-risk threshold (FMMRS ≥8) offered higher specificity (69.7%) but lower sensitivity (57.1, 95% CI 25–84) (four of seven major complications) for major complications; full operating characteristics across all thresholds are provided in Supplementary Table S3.

## 4 Discussion

In this preliminary independent external validation of 40 surgically treated FMMs, the FMMRS showed limited discrimination for both complication endpoints, with confidence intervals that included chance-level performance but overestimated absolute AnyComp risk in the intermediate- and high-risk groups, and with slight underprediction in the low-risk group. Although anatomical classifications have improved surgical description and approach selection, they do not provide individualized, transportable estimates of postoperative complication risk (8, 9, 19). In this context, the FMMRS is the first published tool to translate preoperative MRI anatomy into a practical surgical risk score (3). This study should be read as a preliminary, signal-detection validation rather than a definitive assessment of FMMRS calibration, since with just 14 primary and 7 major events, all performance estimates are imprecise; therefore, our findings are limited to hypothesis-generating. These findings suggest that the FMMRS may, at most, function as a coarse ordinal marker of relative surgical risk. Thus, these published probability estimates may not be reliable for individualized preoperative counseling without recalibration.

The distinction between discrimination and calibration is central to this interpretation. Point estimates indicated limited, broadly similar discrimination across the two outcome definitions: AUC 0.64 for any complication and AUC 0.67 for major complications. However, both confidence intervals included chance-level performance, so chance-level discrimination could not be excluded for either outcome, and neither performance level was sufficient for individualized prediction. These values were lower than those reported in the derivation study, where the FMMRS achieved an AUC of 0.81 in the development cohort and 0.86 in the original external set, compared with 0.64 and 0.67 in our cohort. This difference may reflect variation in case mix, surgical practice, outcome ascertainment, temporal factors, or random variation related to the limited sample size (22, 23). The three score components remain anatomically plausible correlates of operative complexity, reflecting ventral exposure, craniospinal extent, and vertebral artery relationships (3, 7, 9). These components retain face validity as markers of surgical difficulty, but the present data do not establish robust discrimination for individualized prediction. The dissociation between discrimination and calibration is well recognized in external validation studies of clinical prediction models, where rank-ordering tends to be more robust than absolute probability estimates (24, 25). However, calibration failure indicates that the score’s absolute risk estimates were too high for our patients. Critically, this overestimation was not uniform but intensified toward the high-risk end of the score, where predicted probabilities approached certainty, yet observed complications did not. The score was therefore least reliable in the very patients for whom precise risk estimates matter most.

The pattern of apparent miscalibration was not uniform across risk strata, although all observed–expected comparisons are subject to substantial imprecision. In the published high-risk group (FMMRS 8–9), the expected complication probability approached 96%, whereas the observed event rate in our cohort was 42.9% for any complication, a discrepancy of 53.5 percentage points. The direction of overestimation appears consistent: the entire 95% CI for the high-risk observed rate lies below the published expected band. Its magnitude (Δ = −53.5 pp) should be interpreted with caution given the wide observed 95% CI (21.4–67.4%). The calibration slope, intercept, and observed-vs-expected comparisons provide more reliable evidence of calibration in this setting, because predicted probabilities in the high-risk group approach 1 and the Hosmer–Lemeshow statistic is correspondingly inflated and unstable. Score-level trend testing did not reach statistical significance for either AnyComp or major complications, likely because of the limited sample size and sparse event counts. Taken together, these findings suggest that the FMMRS retains some, albeit limited, ordinal risk-stratification capacity in this cohort while overestimating absolute AnyComp risk, most markedly among high-score patients, with the caveat that the magnitude of the discrepancy is imprecise given the wide confidence intervals.

Several factors, likely acting in combination, may explain the observed overestimation. At the model level, the published FMMRS provides predicted risk as score-specific probability ranges rather than an explicit regression equation, and the upper end of the score is anchored by sparse derivation-cohort cells. Only four patients with a score of 9 generated the 96.1–100% probability estimate, rendering the high-score predictions inherently imprecise (3). This imprecision alone does not explain the discrepancy. Even using the full published band rather than its midpoint, the observed high-risk rate and its entire confidence interval stayed below the band’s lower bound. This finding supports overprediction of the published risk mapping in this cohort and suggests that the discrepancy was not solely attributable to midpoint selection. The observed miscalibration may also reflect differences in the case mix. Although our cohort had comparable anatomical complexity based on several FMMRS variables, patients had a higher baseline functional status, smaller tumors, and fewer presenting neurological symptoms; however, the FMMRS does not capture these features but plausibly influences postoperative resilience. In addition, VA involvement was more frequent in our cohort than in the derivation cohort (75% vs. 53%, *p* = 0.049) yet did not translate into proportionally higher complication rates. The FMMRS scores VA involvement as a single binary weight, which may not capture the spectrum from simple contact to complete encasement, nor the surgeon’s capacity to manage it. Differences in surgical expertise, perioperative care, and the clinical interpretation of VA involvement may also contribute to between-center variation in observed complication risk; however, these factors could not be tested directly in the present study (7, 11, 26). Finally, contemporary refinements in microsurgical technique, neurophysiological monitoring, and perioperative critical care may also have attenuated the morbidity historically associated with anatomically complex tumors (27). The temporal pattern within our own cohort is consistent with this explanation. Complication rates fell substantially between the first and second halves of the accrual period despite a comparable distribution of FMMRS scores. All operations were also performed by a single surgeon at a tertiary high-volume skull base center, so the low observed rate in the high-risk group may reflect the outcome achievable in this setting and in the contemporary period rather than the risk expected across centers with differing case volumes. Multicenter validation spanning centers of differing surgical volumes will be needed to establish whether the overprediction we observed is a property of the score or of the setting in which it was tested.

Clinically, our data caution against using the published FMMRS probabilities as absolute risk estimates. Exploratory threshold analyses suggest that the score may have limited utility as a sensitivity-oriented screening tool for major complications: in our cohort, FMMRS ≥6 captured all major complications, whereas the published high-risk threshold of ≥8 was more specific but identified only 4 of 7 major events. For any complication, the Youden-optimal threshold of ≥7 yielded a sensitivity of 85.7% with a specificity of 42.3%, reflecting the score’s coarser performance for this composite outcome. These findings are hypothesis-generated and do not establish a clinical decision threshold. Pending larger validation studies, the FMMRS may be most appropriately viewed as a descriptive measure of preoperative anatomical complexity rather than as a source of individualized absolute complication probabilities. Similar discrepancies between derivation and external validation performance have been reported for other neurosurgical prediction tools, reinforcing the need for independent validation before routine clinical adoption (3, 28).

Although the present findings identify a need for model updating, the development of a deployable recalibrated score is not possible from the published score-specific probability intervals alone. The absence of the original regression equation and patient-level derivation data precludes direct updating of the original model coefficients. Larger multicenter cohorts, ideally including the derivation data, will be needed either to update the published score-to-risk mapping or to re-estimate a new model.

Postoperative complications should not be equated with the absence of clinical benefit. In this cohort, documented improvement of preoperative symptoms was common, including among several patients who experienced AnyComp. Because the symptom trajectory was assessed retrospectively and descriptively, this observation should be interpreted cautiously; nevertheless, it emphasizes that perioperative morbidity and long-term clinical benefit are distinct dimensions of surgical outcome.

To our knowledge, this study represents the first fully independent external validation of the FMMRS, performed by an unrelated investigator team in a geographically and temporally distinct surgical cohort. It is also the first to evaluate the transportability of published absolute-risk estimates using calibration metrics and observed-expected comparisons, complementing the discrimination-only validation reported by the original authors. Reporting was structured in accordance with TRIPOD principles for external validation of prediction models, incorporating discrimination (AUC with 95% CI), calibration (slope, intercept, and Brier score), threshold performance across all candidate cut-points, and trend testing (21). Outcome-blind recalculation of the FMMRS in this cohort minimized assessment bias.

This study has several limitations. It was retrospective and conducted as a single-surgeon series at a high-volume skull base center, which may limit generalizability to settings with different surgical expertise, case selection, and perioperative care. Because a single surgeon performed all cases, the lower observed rates cannot be separated from surgical expertise at this sample size. The sample size was modest, with 14 any-complication events and 7 major-complication events, which limited the precision of discrimination and calibration estimates, and falls well below established sample-size recommendations for external validation of prediction models (29). The AUC confidence intervals include 0.50 at both endpoints, so chance-level discrimination cannot be excluded, and the calibration slope interval ranges from no relationship to moderate agreement. In addition, expected probabilities had to be approximated from published score-specific risk ranges because the original FMMRS model equation was not available. The AnyComp endpoint was ascertained retrospectively and may not have been identical to the outcome definition used in the derivation study, and follow-up duration varied substantially across patients, which may have affected the ascertainment of transient or delayed postoperative events. Retrospective ascertainment of complications may also have introduced classification bias, particularly for transient or minor events. Finally, calibration drift may reflect not only score instability but also temporal improvements in skull-based technique, monitoring, and postoperative care since the derivation cohort (3). The temporal analysis was *post hoc* and cannot separate surgical experience from changes in perioperative care or complication documentation.

## 5 Conclusion

In this preliminary independent external validation, FMMRS point estimates suggested limited discrimination, but uncertainty was substantial, and chance-level performance could not be excluded. The published score-to-risk mapping overestimated AnyComp probabilities in the intermediate- and high-risk groups, particularly among high-score patients. Until large multicenter validation and model updates are available, the FMMRS should not be used to provide individualized absolute estimates of postoperative complication risk.

## Data Availability

The raw data supporting the conclusions of this article will be made available by the authors, without undue reservation.

## Glossary

AnyComp: any postoperative complication
AUC: area under the receiver operating characteristic curve
CI: confidence interval
CS: craniospinal
CSF: cerebrospinal fluid
DL: dentate ligament
FMM: foramen magnum meningioma
FMMRS: Foramen Magnum Meningioma Risk Score
GTR: gross total resection
HL: Hosmer–Lemeshow
IQR: interquartile range
KPS: Karnofsky Performance Status
NPV: negative predictive value
PEG: percutaneous endoscopic gastrostomy
PPV: positive predictive value
ROC: receiver operating characteristic
SC: spinocranial
TRIPOD: Transparent Reporting of a Multivariable Prediction Model for Individual Prognosis or Diagnosis
VA: vertebral artery
WHO: World Health Organization.
