# Supplementary material for: Independent external validation of the foramen magnum meningioma risk score: limited discrimination and overestimation of absolute risk
Source: Front Neurol. 2026 Oct 23;17:1926742. doi: 10.3389/fneur.2026.1926742 (PMC13739601; doi:10.3389/fneur.2026.1926742)
Supplement: Supplementary file 1 [file Table_1.docx]

**Supplementary Table S1. The Foramen Magnum Meningioma Risk Score (FMMRS): component scoring and published complication-risk estimates from the derivation study.**

**(A) Component scoring**

| **Factor** | **Category** | **Points** |
| --- | --- | --- |
| Anteroposterior location (relative to the dentate ligament) | Posterior | 1 |
|  | Anterior | 5 |
| Craniocaudal location | Spinocranial | 1 |
|  | Craniospinal | 3 |
| Vertebral artery involvement | Absent | 0 |
|  | Present | 1 |

**(B) Predicted complication risk by FMMRS total score**

| **Risk class** | **FMMRS score** | **Published predicted risk of complications, % (band)** | **Midpoint used, %** |
| --- | --- | --- | --- |
| Low (2–4) | 2 | 0.00–4.76 | 2.38 |
|  | 3 | 5.31–9.52 | 7.41 |
|  | 4 | 10.50–28.60 | 19.55 |
| Intermediate (5–7) | 5 | 29.30–38.10 | 33.70 |
|  | 6 | 39.20–61.90 | 50.55 |
|  | 7 | 62.40–76.20 | 69.30 |
| High (8–9) | 8 | 77.40–95.20 | 86.30 |
|  | 9 | 96.10–100.00 | 98.05 |

The FMMRS is the sum of three preoperative MRI-based component scores (Panel A) and ranges from 2 to 9 points; totals are grouped as low (2–4), intermediate (5–7), or high (8–9) risk. Published predicted complication-risk bands (Panel B) are those reported in the derivation study (Fatima et al.); the midpoint of each band is the patient-level probability applied in the calibration analyses of the present study. *FMMRS, Foramen Magnum Meningioma Risk Score; MRI, magnetic resonance imaging*

**Supplementary Table S2. Per-patient Foramen Magnum Meningioma Risk Score (FMMRS) components, predicted complication risk, and observed outcomes (n=40).**

| **No.** | **FMMRS component points^*^** | | | **Total FMMRS** | **Predicted risk of complications (%) ^**^** | | | **Risk group** | **Outcomes^***^** | |
| --- | --- | --- | --- | --- | --- | --- | --- | --- | --- | --- |
|  | **AP** | **CC** | **VA** |  | **Midpoint** | **Lower** | **Upper** |  | **Any** | **Major** |
| 1 | 5 | 1 | 1 | 7 | 69.30 | 62.40 | 76.20 | Intermediate | No | No |
| 2 | 5 | 3 | 1 | 9 | 98.05 | 96.10 | 100.00 | High | Yes | No |
| 3 | 1 | 1 | 1 | 3 | 7.41 | 5.31 | 9.52 | Low | No | No |
| 4 | 5 | 1 | 0 | 6 | 50.55 | 39.20 | 61.90 | Intermediate | No | No |
| 5 | 5 | 1 | 1 | 7 | 69.30 | 62.40 | 76.20 | Intermediate | No | No |
| 6 | 5 | 3 | 1 | 9 | 98.05 | 96.10 | 100.00 | High | Yes | Yes |
| 7 | 1 | 1 | 0 | 2 | 2.38 | 0.00 | 4.76 | Low | No | No |
| 8 | 5 | 3 | 1 | 9 | 98.05 | 96.10 | 100.00 | High | Yes | Yes |
| 9 | 5 | 3 | 1 | 9 | 98.05 | 96.10 | 100.00 | High | No | No |
| 10 | 5 | 1 | 1 | 7 | 69.30 | 62.40 | 76.20 | Intermediate | Yes | No |
| 11 | 5 | 1 | 1 | 7 | 69.30 | 62.40 | 76.20 | Intermediate | No | No |
| 12 | 5 | 1 | 1 | 7 | 69.30 | 62.40 | 76.20 | Intermediate | Yes | No |
| 13 | 5 | 1 | 1 | 7 | 69.30 | 62.40 | 76.20 | Intermediate | No | No |
| 14 | 1 | 1 | 0 | 2 | 2.38 | 0.00 | 4.76 | Low | No | No |
| 15 | 5 | 1 | 0 | 6 | 50.55 | 39.20 | 61.90 | Intermediate | No | No |
| 16 | 5 | 1 | 1 | 7 | 69.30 | 62.40 | 76.20 | Intermediate | No | No |
| 17 | 5 | 1 | 1 | 7 | 69.30 | 62.40 | 76.20 | Intermediate | Yes | No |
| 18 | 5 | 3 | 1 | 9 | 98.05 | 96.10 | 100.00 | High | No | No |
| 19 | 5 | 3 | 1 | 9 | 98.05 | 96.10 | 100.00 | High | No | No |
| 20 | 5 | 3 | 0 | 8 | 86.30 | 77.40 | 95.20 | High | No | No |
| 21 | 1 | 3 | 1 | 5 | 33.70 | 29.30 | 38.10 | Intermediate | No | No |
| 22 | 5 | 1 | 1 | 7 | 69.30 | 62.40 | 76.20 | Intermediate | Yes | Yes |
| 23 | 5 | 1 | 1 | 7 | 69.30 | 62.40 | 76.20 | Intermediate | No | No |
| 24 | 5 | 3 | 1 | 9 | 98.05 | 96.10 | 100.00 | High | No | No |
| 25 | 1 | 3 | 1 | 5 | 33.70 | 29.30 | 38.10 | Intermediate | No | No |
| 26 | 5 | 3 | 1 | 9 | 98.05 | 96.10 | 100.00 | High | Yes | No |
| 27 | 1 | 1 | 0 | 2 | 2.38 | 0.00 | 4.76 | Low | No | No |
| 28 | 1 | 3 | 0 | 4 | 19.55 | 10.50 | 28.60 | Low | No | No |
| 29 | 1 | 3 | 0 | 4 | 19.55 | 10.50 | 28.60 | Low | Yes | No |
| 30 | 5 | 1 | 1 | 7 | 69.30 | 62.40 | 76.20 | Intermediate | Yes | Yes |
| 31 | 1 | 3 | 1 | 5 | 33.70 | 29.30 | 38.10 | Intermediate | No | No |
| 32 | 5 | 3 | 1 | 9 | 98.05 | 96.10 | 100.00 | High | No | No |
| 33 | 5 | 3 | 1 | 9 | 98.05 | 96.10 | 100.00 | High | Yes | Yes |
| 34 | 5 | 1 | 0 | 6 | 50.55 | 39.20 | 61.90 | Intermediate | Yes | Yes |
| 35 | 1 | 3 | 1 | 5 | 33.70 | 29.30 | 38.10 | Intermediate | No | No |
| 36 | 5 | 1 | 1 | 7 | 69.30 | 62.40 | 76.20 | Intermediate | Yes | No |
| 37 | 5 | 3 | 1 | 9 | 98.05 | 96.10 | 100.00 | High | No | No |
| 38 | 5 | 3 | 0 | 8 | 86.30 | 77.40 | 95.20 | High | Yes | Yes |
| 39 | 5 | 1 | 1 | 7 | 69.30 | 62.40 | 76.20 | Intermediate | No | No |
| 40 | 5 | 3 | 1 | 9 | 98.05 | 96.10 | 100.00 | High | No | No |

*AP, anteroposterior location relative to the dentate ligament (posterior=1, anterior=5 points); CC, craniocaudal location (spinocranial=1, craniospinal=3 points); VA, vertebral artery involvement (absent=0, present=1 point); FMMRS, Foramen Magnum Meningioma Risk Score.*

*^*^Component points follow the weighting scheme of the derivation study (Fatima et al., Table 4). The total FMMRS equals the sum of the three component points and ranges from 2 to 9.*

*^**^Predicted risk values are the published score-specific probability bands from Table 4 of the derivation study (Fatima et al.). The midpoint is the arithmetic mean of the lower and upper bounds of each band and was the value used for the primary calibration analyses; the lower and upper bounds were retained for the band-based calibration robustness check.*

*^***^Any, any postoperative complication (primary outcome); Major, major complication (secondary outcome). Patient identifiers have been replaced with sequential study numbers; no protected health information is included.*

**Supplementary Table S3. Operating characteristics of the FMMRS by threshold.**

| Outcome/ Cut–point | Sens. %  (95% CI) | Spec. (%) | PPV (%) | NPV %  (95% CI) | Acc. (%) | Youden | Note |
| --- | --- | --- | --- | --- | --- | --- | --- |
| Primary ≥3 | 100.0 (78–100) | 11.5 | 37.8 | 100.0 (44–100) | 42.5 | +11.5 | – |
| Primary ≥4 | 100.0 (78–100) | 15.4 | 38.9 | 100.0 (51–100) | 45.0 | +15.4 | – |
| Primary ≥5 | 92.9 (69–99) | 19.2 | 38.2 | 83.3 (44–97) | 45.0 | +12.1 | ≥Intermediate |
| Primary ≥6 | 92.9 (69–99) | 34.6 | 43.3 | 90.0 (60–98) | 55.0 | +27.5 | Sensitivity-oriented |
| Primary ≥7 | 85.7 (60–96) | 42.3 | 44.4 | 84.6 (58–96) | 57.5 | +28.0 | Youden–optimal |
| Primary ≥8 | 42.9 (21–67) | 69.2 | 42.9 | 69.2 (50–83) | 60.0 | +12.1 | =High risk |
| Primary ≥9 | 35.7 (16–61) | 73.1 | 41.7 | 67.9 (49–82) | 60.0 | +8.8 | – |
| Major ≥3 | 100.0 (65–100) | 9.1 | 18.9 | 100.0 (44–100) | 25.0 | +9.1 | – |
| Major ≥4 | 100.0 (65–100) | 12.1 | 19.4 | 100.0 (51–100) | 27.5 | +12.1 | – |
| Major ≥5 | 100.0 (65–100) | 18.2 | 20.6 | 100.0 (61–100) | 32.5 | +18.2 | ≥Intermediate |
| Major ≥6 | 100.0 (65–100) | 30.3 | 23.3 | 100.0 (72–100) | 42.5 | +30.3 | Youden–optimal |
| Major ≥7 | 85.7 (49–97) | 36.4 | 22.2 | 92.3 (67–99) | 45.0 | +22.1 | – |
| Major ≥8 | 57.1 (25–84) | 69.7 | 28.6 | 88.5 (71–96) | 67.5 | +26.8 | =High risk |
| Major ≥9 | 42.9 (16–75) | 72.7 | 25.0 | 85.7 (69–94) | 67.5 | +15.6 | – |

At each threshold, sensitivity, specificity, positive predictive value (PPV), negative predictive value (NPV), accuracy, and the Youden index (sensitivity+specificity-100) were calculated. 95% confidence intervals (Wilson method) are reported for sensitivity and NPV. “≥Intermediate" and “=High risk” denote the published FMMRS thresholds (≥5 and ≥8); “Youden-optimal” maximizes the Youden index; “Sensitivity-oriented” is a neighboring threshold with a Youden index within 1 percentage point of the maximum and higher sensitivity. All threshold metrics are exploratory and rest on 14 any-complication and 7 major-complication events; confidence intervals are wide, and these cut-points require prospective confirmation. *Sens.=sensitivity; Spec.=specificity; Acc.=accuracy; CI=confidence interval; FMMRS=Foramen Magnum Meningioma Risk Score.*

**Supplementary Table S4. Comparison of case mix and postoperative outcomes between the first and second halves of the accrual period.**

|  | **2006–2015 (n=16)** | **2016–2025 (n=24)** | **p** |
| --- | --- | --- | --- |
| **Case mix** |  |  |  |
| FMMRS total, median (IQR) | 7 (7–9) | 7 (5–9) | 0.29 |
| High-risk group (FMMRS 8–9), n (%) | 6 (37.5) | 8 (33.3) | 1.00 |
| **Postoperative outcomes** |  |  |  |
| Any postoperative complication, n (%) | 11 (68.8) | 3 (12.5) | 0.0005 |
| Major complication, n (%) | 6 (37.5) | 1 (4.2) | 0.011 |
| Gross total resection, n (%) | 11 (68.8) | 20 (83.3) | 0.44 |
| Length of stay, days, median (IQR) | 8 (5–12) | 4 (3–4) | 0.0004 |
| **High-risk group (FMMRS 8–9)** |  |  |  |
| Observed complication rate, n/N (%) | 4/6 (66.7) | 2/8 (25.0) | – |
| Expected complication rate (%) * | 96.1 | 96.6 | – |
| Observed – expected (percentage points) | –29.4 | –71.6 | – |

Values are n (%) unless otherwise stated. Categorical variables were compared using Fisher's exact test and continuous variables using the Mann-Whitney U test. This analysis was performed post hoc, was not prespecified, and used an arbitrary division of the accrual period at its midpoint; the p values are therefore exploratory and are presented for descriptive purposes only.

**Expected complication rates are the means of the published score-specific midpoint probabilities of the derivation study for the patients within each subgroup.*

*IQR, interquartile range; FMMRS, Foramen Magnum Meningioma Risk Score.*
